# Stable Prenucleation Calcium Carbonate Clusters Define Liquid–Liquid Phase Separation

**DOI:** 10.1002/anie.201915350

**Published:** 2020-02-25

**Authors:** Jonathan T. Avaro, Stefan L. P. Wolf, Karin Hauser, Denis Gebauer

**Affiliations:** ^1^ Department of Chemistry University of Konstanz Universitätsstrasse 10 78457 Konstanz Germany; ^2^ Present address: Institute of Inorganic Chemistry Leibniz University of Hannover Callinstrasse 9 30167 Hannover Germany

**Keywords:** calcium carbonate, liquid–liquid phase separation, nonclassical nucleation, phase diagram, prenucleation clusters

## Abstract

Liquid–liquid phase separation (LLPS) is an intermediate step during the precipitation of calcium carbonate, and is assumed to play a key role in biomineralization processes. Here, we have developed a model where ion association thermodynamics in homogeneous phases determine the liquid–liquid miscibility gap of the aqueous calcium carbonate system, verified experimentally using potentiometric titrations, and kinetic studies based on stopped‐flow ATR‐FTIR spectroscopy. The proposed mechanism explains the variable solubilities of solid amorphous calcium carbonates, reconciling previously inconsistent literature values. Accounting for liquid–liquid amorphous polymorphism, the model also provides clues to the mechanism of polymorph selection. It is general and should be tested for systems other than calcium carbonate to provide a new perspective on the physical chemistry of LLPS mechanisms based on stable prenucleation clusters rather than un‐/metastable fluctuations in biomineralization, and beyond.

## Graphical abstract

**A quantitative, general model** for liquid–liquid phase separation is introduced based on “nonclassical” nucleation via prenucleation clusters. Potentiometric titrations and stopped‐flow ATR‐FTIR spectroscopy verify the predictive power of the model for the aqueous calcium carbonate system. This model reconciles the previously inconsistent literature values for the solubilities of amorphous calcium carbonates.

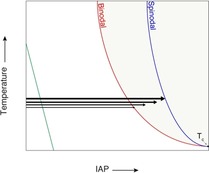

Liquid–liquid phase separation (LLPS) occurs in cells,^1^ plays a role during infections,^2^ and is fundamental to amphiphilic self‐assembly processes.^3^ Liquid–liquid‐separated precursor species, on the other hand, also form in inorganic systems, for example, in oxide melts^4^ and aqueous salt solutions. In the latter case, the most abundant biomineral,^5^, ^6^ calcium carbonate, is the prime example.^7^ While so‐called “polymer‐induced liquid precursors” (PILPs) of calcium carbonate were first described in the late 1990s,^8^ it has become evident that these species constitute polymer‐stabilized rather than polymer‐induced states, since they also occur in purely inorganic systems.^9^, ^10^, ^11^ As a result of their major role in controlling crystal morphologies in biomineralization,^12^ research activities on such liquid and solid amorphous intermediates of CaCO_3_ are intensive.^13^, ^14^, ^15^, ^16^, ^17^ Indeed, numerous studies have impressively illustrated their great use for controlling calcium carbonate crystallization also in vitro.^7^, ^18^, ^19^, ^20^, ^21^, ^22^, ^23^

However, the mechanisms by which liquid or solid amorphous intermediates form remain under debate.^24^ Recent studies^25^, ^26^ suggested that the notions of classical nucleation theory would sufficiently explain experimental observations upon precipitation from the metastable zone of the phase diagrams, that is, close to the binodal limit. Furthermore, it was proposed that PILPs would actually not be liquid, but consist of polymer‐interconnected solid nanoparticles.^14^ On the other hand, spinodal decomposition—that is, barrier‐less precipitation from the unstable region of phase diagrams—was identified as a mechanism towards mineral droplet formation.^27^ The corresponding notions of Cahn and Hilliard^28^ were employed for the derivation of a phase diagram of the aqueous calcium carbonate system by Zou et al.,^29^ which exhibited an upper critical solution temperature, in contrast to previous models.^27^, ^30^ Despite the explanatory power of the so‐called “prenucleation cluster (PNC) pathway”,^31^, ^32^ sometimes referred to as “nonclassical nucleation”, however, a corresponding quantitative theory for phase separation in the aqueous calcium carbonate system is still lacking.

Here, we developed a model where ion association thermodynamics in the homogeneous phase, based on the PNC model, determines the liquid–liquid miscibility gap of the aqueous calcium carbonate system. We verified this model experimentally, using potentiometric titrations and stopped‐flow ATR‐FTIR spectroscopy. The mechanism explains the variable solubilities of solid amorphous calcium carbonates from different regions of the metastable zone of the liquid–liquid phase diagram. This reconciles previously inconsistent literature values,^25^, ^33^, ^34^ while the model defines a lower‐critical solution temperature of the liquid–liquid miscibility gap. Since it also accounts for liquid–liquid amorphous polymorphism,^35^ it may provide clues to the mechanism of polymorph selection.^36^ Altogether, the model offers a novel physical chemical perspective on the phenomenon of LLPS based on stable PNCs rather than un‐/metastable fluctuations. The model is general and should be tested for other systems in the future.

The experimentally observed calcium carbonate ion association (SI, Figure S1) is consistent with the formation of PNCs,^34^ as opposed to alternative models (see Sections 1 and 2 in the Supporting Information for details).^26^ According to the notions of the so‐called PNC pathway, which was introduced in detail elsewhere,^32^ solute PNCs can become phase‐separated nanodroplets upon crossing the corresponding liquid–liquid binodal limit because of a decrease in the dynamics upon increasing the calcium and carbonate coordination numbers within PNCs. This means, in contrast to classical nucleation theory^37^ and existing models of spinodal decomposition,^28^ thermodynamically stable populations of ion associates, rather than un‐ and metastable fluctuations, serve as fundamental precursors to the new phase.^24^ Previous assessments of the dynamics of the hydrogen‐bond network of water by means of THz absorption spectroscopy revealed a distinct change in the prenucleation solution upon crossing the solubility threshold for amorphous calcium carbonates (ACCs) forming under these conditions.^11^ This must be due to LLPS, being consistent with the proposed change in the (solution) dynamics underlying phase separation from PNCs.^27^, ^32^ Thus, potentiometric titrations allow the quantitative determination of the ion activity product (IAP) defining the liquid–liquid binodal limits in a temperature range of 15–45 °C in terms of the solubilities of the initially formed ACCs (Table S1). According to previous studies, proto‐calcite (pc) and proto‐vaterite (pv) ACC form at pH 9.00 and pH 10.0, respectively,^34^, ^38^ and proto‐aragonite (pa) ACC at pH 10.0 above about 35 °C.^39^

Previous studies also suggested that these ACCs form by the dehydration and solidification of the corresponding dense liquids.^11^ Since the dense liquids form by liquid–liquid demixing, which occurs with a certain finite probability upon crossing the liquid–liquid binodal limit, increasing the rate of mixing of calcium and carbonate solutions will allow entry into the metastable zone of the liquid–liquid miscibility gap deeper and deeper before it occurs (Figure 1). If the proposed mechanism is true, ACC solubility should thus progressively also increase as the mixing rate increases, which is indeed observed (Figures S2 and S3). ATR‐FTIR spectra of ACCs quenched in ethanol at the slowest and fastest addition rates at pH 9.00 reveal that the proto‐structure remains pc‐ACC and suggest that the observed higher solubilities are due to increased water contents (Figure S4). The highest possible metastability of dense liquids is reflected by the liquid–liquid spinodal limit (Figure 1). For the mechanism of solid ACC formation by dehydration and solidification of liquid precursors, this categorically implies that the liquid–liquid spinodal limit presents an upper limit for ACC solubility. Since it was not possible to realize sufficiently high addition rates in the titration assay to assess this limit, we implemented a direct mixing of more concentrated solutions with concurrent measurements of IAPs. The measured ACC solubilities (Figure 2 A, see also Figure S5) indeed reach a maximum, which represents the liquid–liquid spinodal limit (Table S3). It is in excellent agreement with the value of ACC solubility previously established by Brečević and Nielsen.^33^ Thus, here, we show that as a consequence of their formation mechanism from liquid precursors, ACCs have variable solubilities, where the highest possible one is obtained from the mixing of calcium and carbonate solutions with concentrations in the upper 100 mm regime, which is a common literature value.^25^, ^35^ The mechanism of ACC formation by dehydration of dense liquid precursors thereby reconciles the differing literature values of ACC solubilities in between the binodal and spinodal limits described herein (Tables S1 and S3).

**Figure 1:**
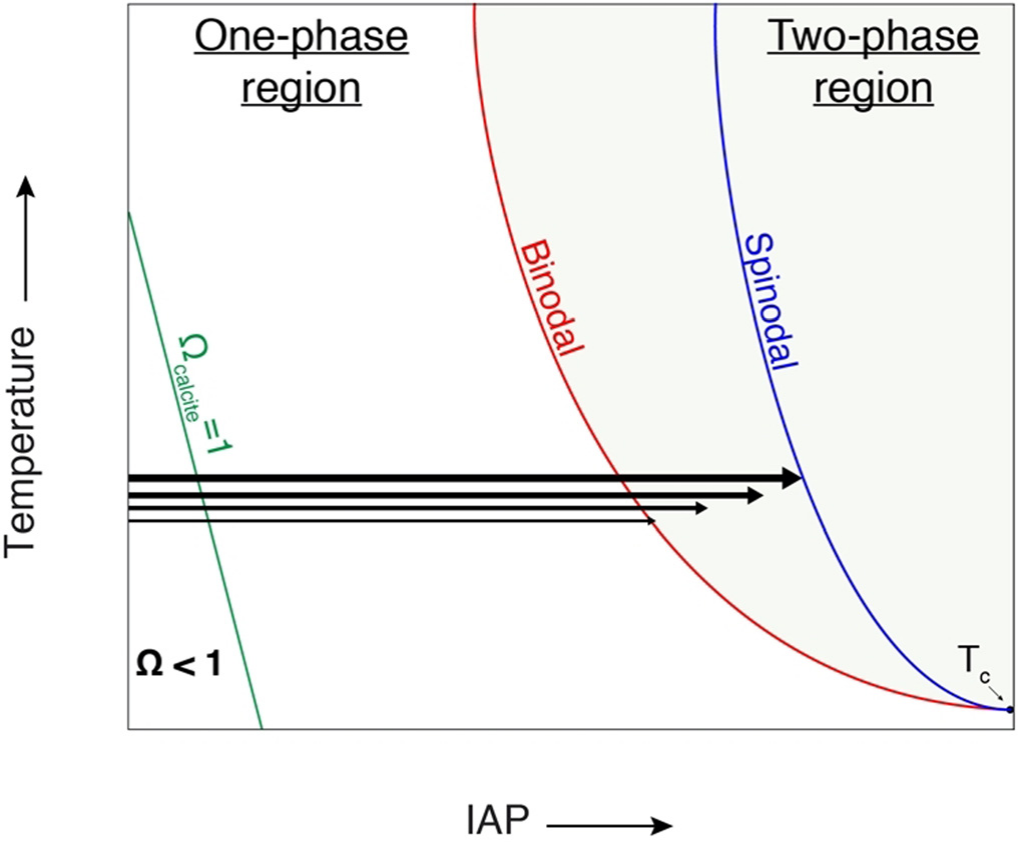
Schematic illustration of the explanation of the observed rate dependency of ACC solubility. As proposed previously,^27^, ^30^ a submerged liquid–liquid miscibility gap is located in the metastable zone of the solid–liquid phase diagram (that is, at ion activity products (IAPs) corresponding to supersaturation ratios *Ω*>1 with respect to the stable solid, calcite). The liquid–liquid phase diagram exhibits a lower critical solution temperature *T*_c_. For the sake of clarity, only the calcite solid–liquid (S‐L) binodal curve is shown, and the right branch of the liquid–liquid miscibility gap defining the compositions of dense liquids is left out. Higher addition rates (bold arrows crossing the liquid–liquid binodal limit pointing from left to right) allow entry into the liquid–liquid miscibility gap to a greater extent than slow rates (corresponding to finer arrows) before demixing occurs, thus yielding more metastable dense liquids. When solid ACCs are subsequently formed by dehydration and solidification of the as‐formed precursor dense liquids, their solubilities directly reflect the metastability of the liquid precursors. Increasingly fast mixing (illustrated by progressively bolder arrows) of calcium and carbonate solutions will thus provide access to more and more metastable solid ACCs, with higher and higher solubilities. In this mechanism, the highest possible metastability of the dense liquid precursor, and, with it, the highest solubility of ACC, is defined by the spinodal limit.

**Figure 2:**
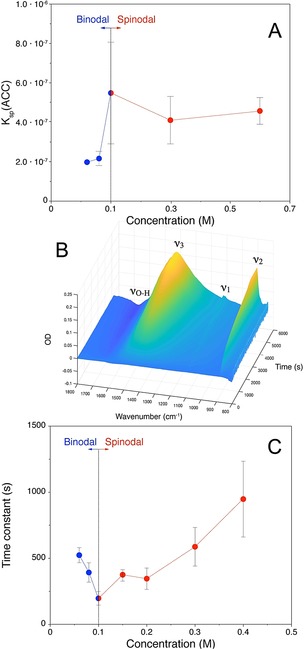
A) Evolution of ACC solubility *K*_sp_(ACC), determined by direct mixing of reactant solutions and potentiometric measurements, as a function of the initial reactant concentration of calcium and carbonate solutions. The significant step indicates entering the liquid–liquid spinodal regime. B) Temporal evolution of carbonate vibrational modes (ν_1_, ν_2_, ν_3_ with combination mode) and OH‐bending mode in water (ν_O‐H_) after mixing of CaCl_2_ (0.3 m) and Na_2_CO_3_ (0.3 m) solutions at a 1:1 volume ratio. The water mode becomes negative with time as calcium carbonate forms and precipitates onto the internal reflection element of the ATR cell, thereby replacing water molecules. C) Dependence of the time constant (determined for the kinetics of calcium carbonate formation based on the time development of the ν_2_ carbonate out‐of‐plane vibrational band, Figure S5) on the initial calcium and carbonate concentration prior to mixing. The minimum value in the obtained time constant at an initial concentration of 0.1 m shows that the reaction kinetics become fastest at this point, as expected for the spinodal limit. Indeed, this point corresponds to the liquid–liquid spinodal limit determined in the potentiometric titrations within experimental certainty (cf. A).

To independently validate the locus of the spinodal limit obtained from potentiometric data in direct mixing experiments, we performed a kinetic analysis using liquid‐state ATR‐FTIR spectroscopy.^40^, ^41^ The data show the evolution of the characteristic carbonate as well as water vibrational bands (Figure 2 B) after mixing. Depending on the concentrations of the solutions prior to mixing, the time transients of the normalised carbonate ν_2_ vibrational band present distinct kinetics to reach a plateau (Figure S6), which was fitted to a generic model. The as‐obtained time constants exhibit a minimum at an IAP, which was also identified as the spinodal limit in the potentiometric measurements (Figure 2 A). This is fully consistent, as the barrier for phase separation vanishes at the spinodal limit, and the kinetics for the formation of the calcium carbonate are expected to become fastest. The progressively decreasing kinetics upon exceeding the spinodal limit then is likely due to the high viscosities of the formed gel‐like precipitates in this region of the phase diagram (apparent by a strong stirring vortex disappearing after mixing the calcium and carbonate solutions and gradually re‐forming with ongoing precipitation, Movie S1).

The spinodal limit can be quantitatively predicted based on ion association thermodynamics when assessed from the PNC model (see Discussions 3 in the Supporting Information for an explicit deduction). Specifically, the macroscopically accessible ion association constant *K*(cluster) defines the IAP of the spinodal limit, IAP(spinodal), according to Equation (1).

(1) $$\mathrm{IAP}\left(\mathrm{spinodal}\right)=\left[K\left(\mathrm{cluster}\right)\right]{}^{-2}$$

On the other hand, an assessment of the probability for liquid–liquid demixing versus that for the direct formation of crystalline polymorphs (see Discussions 3 in the Supporting Information) allows us to conjecture that the corresponding binodal limit IAP(binodal) is accessible from Equation (2);

(2) $$\mathrm{IAP}\left(\mathrm{binodal}\right)=A\left(\mathrm{polymorph}\right)\;K{}_{\mathrm{sp}}\left(\mathrm{polymorph}\right)\;\ln K\left(\mathrm{cluster}\right)$$

where *A*(polymorph) is a constant and *K*_sp_(polymorph) is the solubility of the different polymorphs, calcite, aragonite, and vaterite. The theoretical spinodal and binodal curves can be calculated from the *K*_sp_(polymorph) values and their temperature dependency from the literature,^42^ as well as the pH and temperature dependency of *K*(cluster) (Table S2). To that end, *A*(calcite)=1.33, *A*(aragonite)=0.98, and *A*(vaterite)=0.39 [Eq. (2)] were first determined by inserting the experimentally determined IAP at 35 °C, where *K*(cluster) is observed to be independent of the pH value.

The as‐calculated theoretical binodal and spinodal curves are in very good agreement with the respective experimental values at pH 9.00 and pH 10.0 (Figure 3). The experimental uncertainty in the spinodal limit is too high to resolve the phenomenon of liquid–liquid polyamorphism, which is fully covered by the model. However, the model does reduce the complex metastable liquid–liquid coexistence to the pH value and temperature dependency (Table S2) of the ion association constant *K*(cluster) and those of the solubilities of the different polymorphs. The model shows that the thermodynamic stability of PNCs not only quantitatively determines the stability (and with it the proto‐structure) of the phase‐separated dense liquid intermediates, but, together with the solubilities of the crystalline forms, also the IAP values, at or beyond which liquid–liquid separation can (binodal limit) and must (spinodal limit) occur. Taken together, the model and data suggest the existence of a triple point of pc‐, pv‐, and pa‐structured dense liquids at about 35 °C, IAP≈2.78×10^−8^, and in between pH 9.00 and 10.0 (Figure 3). Furthermore, the model allows the critical temperature of the liquid‐liquid miscibility gap to be calculated (see Discussions 3 in the Supporting Information), which is well below 100 K for each polymorph, that is, in the experimentally inaccessible region of the aqueous, liquid phase diagrams. In any case, the ion association thermodynamics (Table S2) thereby stipulate a lower critical solution temperature as opposed to other studies.^29^

**Figure 3:**
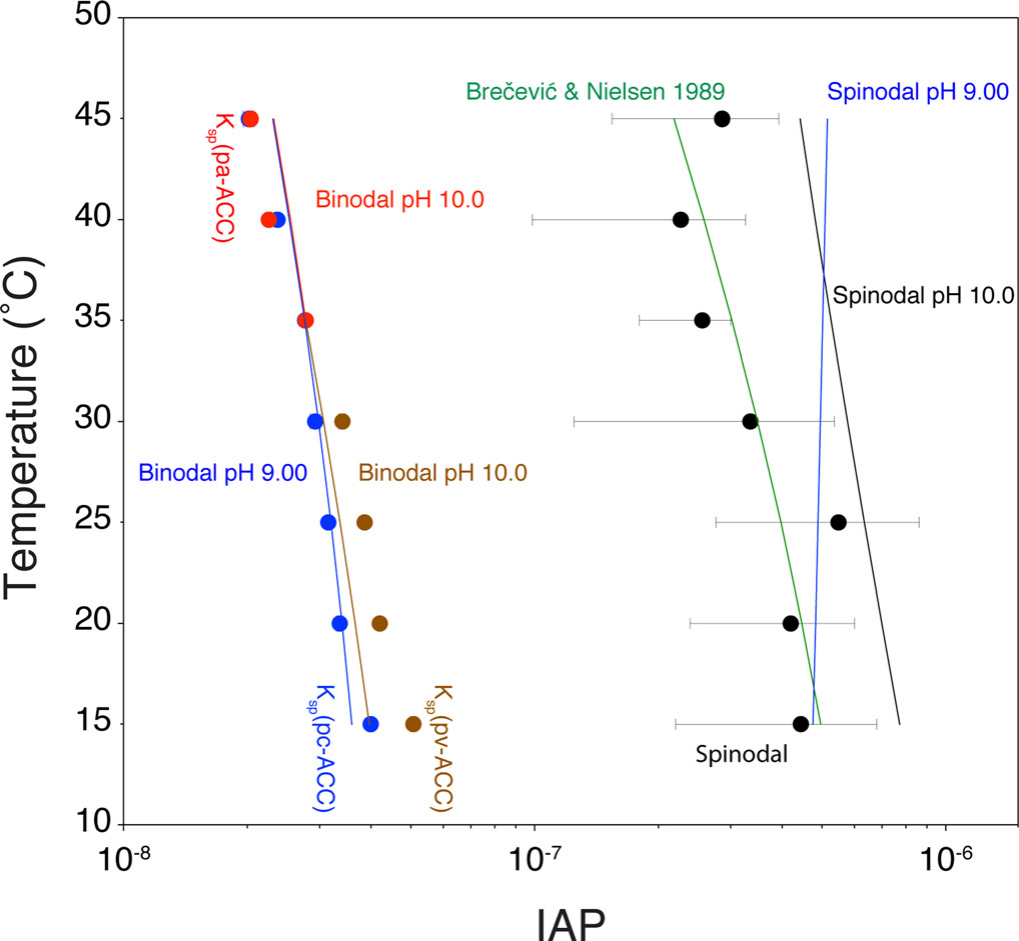
Comparison of measured (data points) and theoretical binodal and spinodal limits (lines) defining the liquid–liquid miscibility gap of the aqueous calcium carbonate system, with pH‐dependent liquid–liquid amorphous polymorphism. Blue and brown data points illustrate experimentally determined binodal limits at pH 9.00 and pH 10.0 below 35 °C, respectively. Red data points show the experimentally determined binodal limit at pH 10.0 above 35 °C, and black data points show the experimentally determined spinodal limit at pH >10.0. The green line represents the solubility of ACC from the literature.^33^ Theoretical binodal and spinodal limits can be calculated from Equations (2) and (1) (derivation: see Discussions 3 in the Supporting Information), respectively, using the standard enthalpies and entropies compiled in Table S2, as well as the temperature dependency of the solubilities of the different polymorphs from the literature.^42^ The blue lines illustrate the theoretical binodal and spinodal curves at pH 9.00, the brown and red lines give the theoretical binodal limit at pH 10.0, accounting for proto‐calcite (pc), proto‐vaterite (pv), and proto‐aragonite (pa) ACCs with the distinct solubilities of the corresponding polymorphs, respectively. The black line represents the theoretical spinodal curve for pH 10.0, which should be compared to the experimental values obtained at high pH values. The blue line on the right is the theoretical spinodal limit at pH 9.00.

In conclusion, we present a “nonclassical”, quantitative model that essentially reduces complex liquid–liquid phase behaviors to the thermodynamics of solute association and the solubilities of crystalline polymorphs. Notably, the corresponding experimentally accessible parameters, specifically, *K*(cluster), *K*_sp_(polymorph), and *A*(polymorph), can be affected by the presence of additives,^43^ the effects of which on LLPS become thereby predictable. Whether or not the model provides a fully quantitative predictive power of such additive effects on LLPS remains to be explored in the future. For the additive‐free scenarios presented here, the model was thoroughly tested for the long‐debated calcium carbonate system, where “classical” nucleation barriers are formidable as the miscibility gap is entered.^11^, ^44^ Classical nucleation theory should, thus, not be considered for the aqueous calcium carbonate system, or likely any system, where homogeneous phase self‐association is significant. In essence, the agreement between the experimental and theoretical values for spinodal and binodal limits (Figure 3) strongly suggests that the fundamental assumption underlying the PNC model (see Sections 1 and 2 in the Supporting Information), that is, that all steps in ion association towards PNCs can be considered to be equal and independent, is indeed fulfilled. Furthermore, our results demonstrate that solid ACCs form by dehydration of the liquid precursors, rather than “classical” nucleation events within dense liquid droplets, because only the former mechanism can rationalize ACC solubilities that are dependent on the mixing rate. Note that, as solid ACCs form by secondary processes, that is, dehydration and concurrent growth, aggregation, and/or coalescence from dense liquid precursors, the size and morphology of solids do not necessarily reflect whether initial phase separation occurred by binodal or spinodal processes. The model should generally be applicable for systems where solute association is driven by the release of hydration water molecules from monomeric chemical constituents^45^ yielding PNCs with a chain‐like structural form, that is, “dynamically ordered liquid‐like oxyanion polymers”, DOLLOPs.^46^ The model provides a fundamentally improved, fully quantitative understanding of LLPS based on thermodynamically stable associated states, PNCs, forming in homogeneous solutions as fundamental precursors to phase separation that may be relevant for corresponding problems in biology as well as inorganic and organic chemistry, and beyond.

## Conflict of interest

The authors declare no conflict of interest.

## Supporting information

As a service to our authors and readers, this journal provides supporting information supplied by the authors. Such materials are peer reviewed and may be re‐organized for online delivery, but are not copy‐edited or typeset. Technical support issues arising from supporting information (other than missing files) should be addressed to the authors.

Supplementary

Supplementary
