# Supplementary material for: Stable Prenucleation Calcium Carbonate Clusters Define Liquid–Liquid Phase Separation
Source: Angew Chem Int Ed Engl. 2020 Feb 25;59(15):6155–9. doi: 10.1002/anie.201915350 (PMC7187218; doi:10.1002/anie.201915350)
Supplement: Supplementary file 1 — Supplementary [file ANIE-59-6155-s001.pdf]

Angewandte Chemie
Eine Zeitschrift der Gesellschaft Deutscher Chemiker

Supporting Information

Stable Prenucleation Calcium Carbonate Clusters Define Liquid–Liquid Phase Separation

Jonathan T. Avaro, Stefan L. P. Wolf, Karin Hauser, and Denis Gebauer**

anie_201915350_sm_miscellaneous_information.pdf
anie_201915350_sm_movie_S1.mp4

Supporting Information

Contents

Experimental Section 1
Supplementary Figures 5
Supplementary Tables 11
Supplementary Discussion 1 13
Supplementary Discussion 2 18
Supplementary Discussion 3 21
References 27

Experimental Section

Titration experiments. A solution of calcium chloride (0.01M) was slowly dosed (0.01mL/min) into sodium carbonate solution using a Titrando 951 titration device operating two Dosino 800 dosing units. The temperature was maintained constant at the indicated values using a double-walled titration vessel (Metrohm 6.1418.250), through which oil was circulated utilizing a thermostat regulated by an external sensor Julabo FP50. The titration vessel was closed with a lid to further aid avoiding any temperature variations, CO_2 in- or outgazing from the buffer, and water evaporation. The titration setup was controlled by the software Tiamo version 2.3, which allows simultaneous and precise dosing of reactant solutions, controlling pH and reading out the voltage of the electrodes. For each measurement, the titration vessel was filled with 50 mL of 10 mM carbonate buffer and brought to the desired temperature. The pH was initially set by mixing adequate ratios of 10 mM sodium carbonate (Sigma-Aldrich 31432-1kg-R) and 10 mM sodium bicarbonate (Emsure 1.06329.1000). After the target temperature was achieved, the pH was finely re-adjusted to the accurate value by adding small increments of 10 mM sodium hydroxide solution (Alfa Aesar 35620). Two additional sets of experiments were performed at higher addition rates of 0.1 and 1 $mL\cdot min^{-1}$. After each experiment, the titration vessel and electrodes tips were thoroughly washed with acetic acid (10%), Milli-Q water and dried with

dust—free tissue paper. Calcium potential and pH were recorded using a polymer-membrane-based calcium ion selective electrode (ISE, Metrohm 6.0508.110) and glass electrodes (Metrohm Unitrode flat membrane 6.0256.100) with internal reference, respectively. The internal reference system of the pH electrode was also used as reference for the calcium ISE. Added volumes and potentials were automatically recorded every 10 seconds during the titration experiments. The calcium ISE was calibrated by titration of 10 mM calcium chloride solution into ultrapure water set to the desired temperature and pH (previously adjusted by addition of NaOH) while a gentle stream of nitrogen was flushed over the calibration sample to avoid CO_2 uptake. A three-point calibration of the pH electrodes was performed using standard pH buffer solutions from Mettler-Toledo with the product numbers: pH = 4.01: 51302069; pH = 7.00: 51302047; pH = 9.21: 51302070.

Potentiometric assessment of the spinodal regime. The spinodal regime was probed via direct mixing of 50 mL calcium chloride solution (0.4 M) with 50 mL sodium carbonate solution (0.4 M). The Carbonate solution in the vessel was rigorously stirred and initially adjusted to pH 11.0. Calcium chloride solution was added directly into the vessel. The calcium potential and pH upon mixing were measured as described above. Due to the formation of a gel, the magnetic stirrer was replaced by a vertical mixer (Metrohm 2.804.0040) with a propeller of 94 mm in diameter (Metrohm 6.1909.010). The stirring speed was adjusted to assure formation of a vortex and optimal mixing but avoid the formation of bubbles affecting the electrode signals. During the experiments, the pH was kept constant via automatic counter titration of sodium hydroxide (1M) and hydrochloric acid (1M). Calibration of the free calcium concentration was performed in ionic strength adjusted environment as described in the literature and activity effects due to increased ionic strength were considered using the Davies equation.[1]

The data evaluation of titration experiments was carried out as described in the literature[1,2] to calculate the IAP values and microscopic and macroscopic binding equilibrium constants (supplementary discussions 1 and 2).

Solid state ATR-FTIR: ATR-FTIR spectra of precipitated ACC were recorded on a Perkin Elmer spectrometer 100 equipped with a diamond ATR crystal from 760 to 4000 cm^{-1} with a spectral resolution of 4 cm^{-1} allowing the detection of the characteristic calcium carbonate bands noted ν_1, ν_2, ν_3 and ν_4 corresponding to the symmetric stretch, out-of-plane bending, asymmetric stretch and in-plane bending vibrational modes, respectively.

Liquid state ATR-FTIR. Time-resolved IR measurements provide insights into kinetic reaction mechanisms as we have shown previously with various applications.[3-5] In this study we performed rapid-scan measurements using a Bruker vertex 80V FTIR spectrometer equipped with a photoconductive mercury cadmium tellurium (MCT) detector (Kolmar Technology) and an ATR diamond single reflection unit (Golden Gate™ Heated Diamond ATR, Specac GS10540) and ZnSe lenses, restricting the spectral range at a lower wavenumber of 800 cm^{-1}, thus the carbonate ν_4 spectral region was not accessible. A custom-made ATR-FTIR stopped-flow with a mixing cell 50 µL (TgK Scientific SF-61/FT-IR) was mounted on top of the heated diamond window. The temperature was kept constant at 25˚C during the experiments using a built-in temperature controller. A Lauda thermostat (Ecoline E300) was used for pre-heating reactants within the umbilical tubes linking a syringe pump to the ATR-FTIR stopped-flow mixing cell on top of the ATR-FTIR unit. Calcium chloride, $CaCl_2$, and sodium carbonate (Na_2CO_3) (0.4M, 0.3M, 0.2M, 0.1M, 0.08M, 0.06M) were mixed at a 1:1 volume ratio within the ATR-FTIR stopped-flow mixing cell. After reactant mixing 100 scans were recorded in rapid-scan mode between 800-1900 cm^{-1} with a scanner velocity of 320 Hertz and a spectral resolution of 4 cm^{-1} and averaged. A waiting time of one second was set in-between each of

the scans. This procedure was repeated 5000 times. Averaged spectra were calculated and off-set corrected between 889-891 cm^{-1}, where no absorption band occurs. The transients were extracted at 869 cm^{-1} corresponding to the second derivative minimum of the ν_2 carbonate vibrational band. In order to compare the reaction kinetics, all transients were normalised to their maximum plateau values reached at the end of each experiment.

Fitting of ATR-FTIR time transients. The equations[6] used for fitting of the transients consist of a combination of at least one exponential growth and one logistic growth regimes as follows:

$$I_{fit}(t) = \alpha \cdot e^{\frac{t}{\tau}} + \frac{K_1}{1 + e^{-F_i \cdot (t - B_i)}} + y_0$$

where $I_{fit}(t)$ is the fitted IR intensity over time; α the inverse of the exponential growth amplitude factor; τ the time constant; K_i the logistic growth saturation value in arbitrary units for level i in this regime, i.e. the heights of each single plateau once the growth rate is minimum; F_i accounting for an evolution of the saturation time (ΔR_i in seconds) from 10% to 90% for level i defined as

$$\Delta R_i = {}^{\ln{(81)}}/_{F_i}$$

according to ref.[6]; B_i the mid-point of growth for level i in seconds; and y_0 the offset.

For all measurements, the same fitting procedure was performed using the built-in Curve Fitting app in Matlab™ (using the bi-square weight method of nonlinear least-squares regression). It first consisted of fitting $I_{fit}(t)$ for n=1 and i=1. If fitting procedure did not converge or reach the maximum iteration number set at 10 000, a second level of logistic growth was added to the fit equation – such as n = 1 and i = 2. Addition of a second level was considered successful if the Pearson product-moment correlation coefficient (r^2) increased by at least 10%.

Supplementary Figures

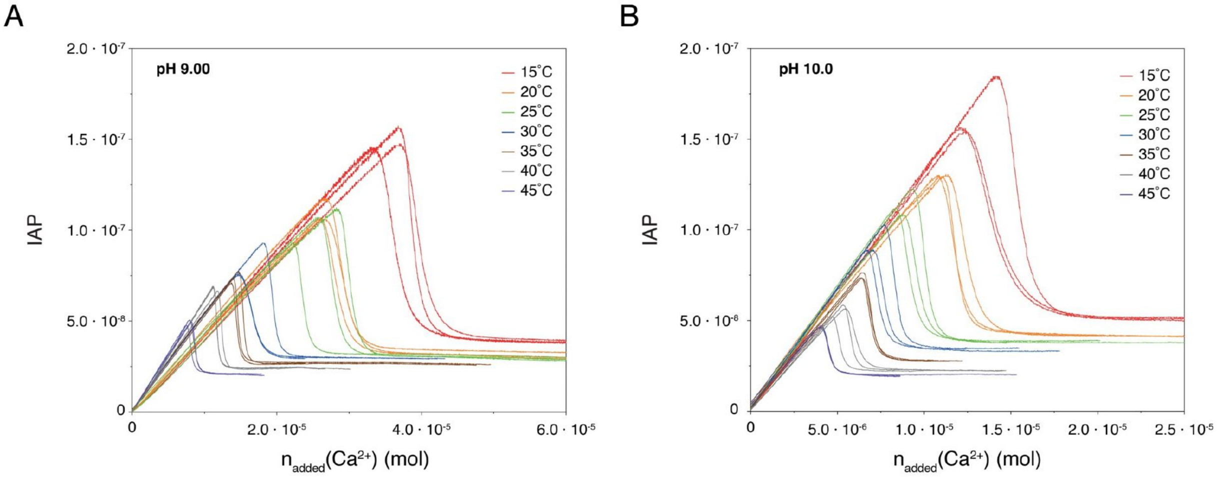

Figure S1. Temperature dependent potentiometric titrations. Calcium chloride solution (0.01M) was added to sodium carbonate buffer (0.01M) at a rate of 0.01 mL/min, while the pH was maintained at a constant level (A: pH 9.00, B: pH 10.0) by automatic counter-titration with 10 mM NaOH. The initial linear increase in ion activity product (IAP) occurs in thermodynamic equilibrium and allows assessing pre-nucleation ion association thermodynamics.[2] At pH 10.0 (B), the prenucleation slopes do not vary significantly with temperature, but they seem to increase with increasing temperature at pH 9.00 indicating that the temperature dependence of ion association is distinct at the two pH levels. Upon nucleation of the solid, the IAP drops and assumes a constant threshold, corresponding to the solubilities of proto-calcite amorphous calcium carbonate (A) and proto-vaterite amorphous calcium carbonate (B).[7] These solubilities identify the liquid-liquid binodal limit, as established by means of THZ spectroscopy elsewhere.[8] At both pH values, the liquid-liquid binodal limit decreases with increasing temperature. Corresponding values are compiled in Table S1.

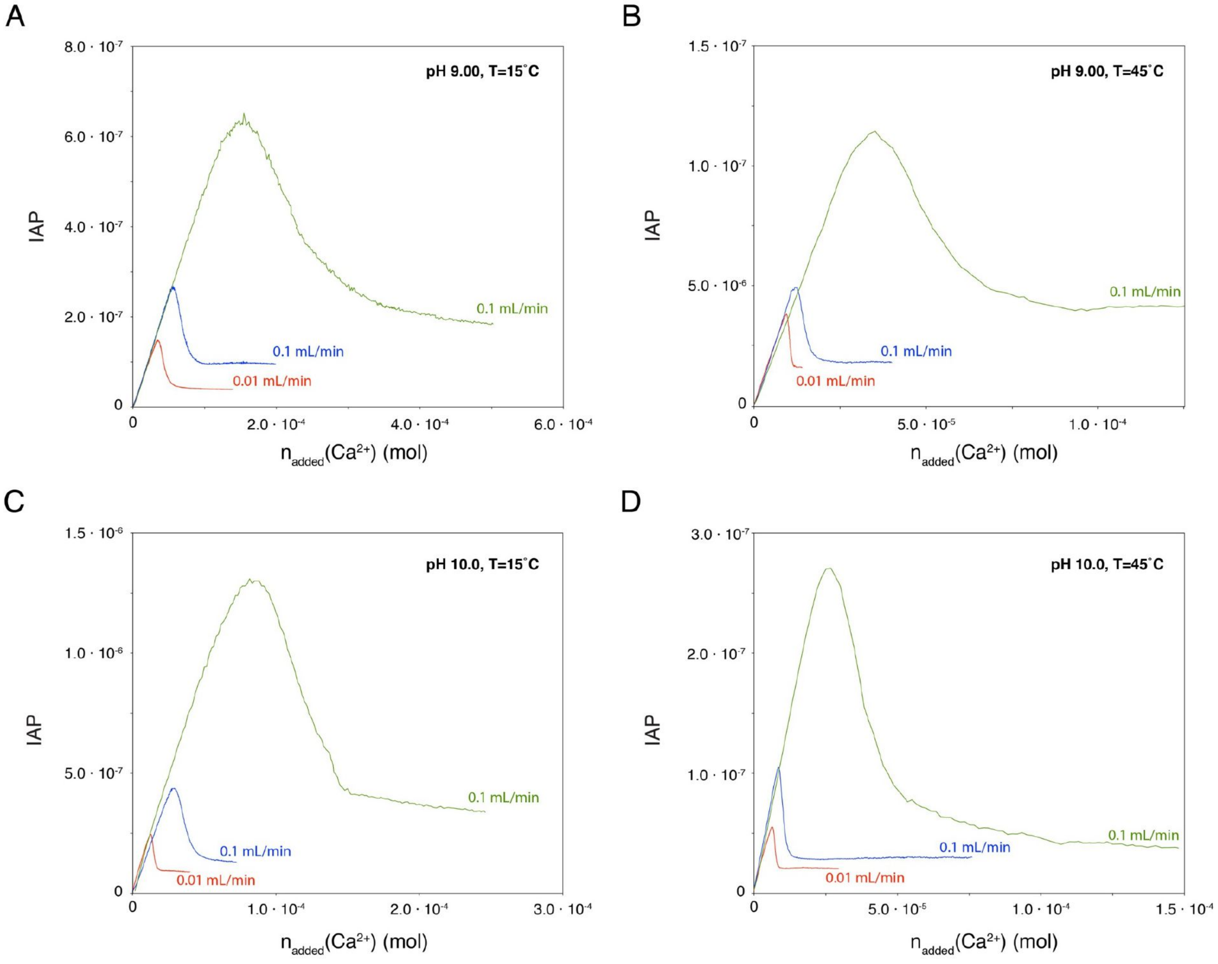

Figure S2. Rate dependent potentiometric titrations. Calcium chloride solution (0.01M) was added to sodium carbonate buffer (0.01M) at different rates as indicated. **A**: pH 9.00, T=15˚C; **B**: pH 9.00, T=45˚C; **C**: pH 10.0, T=15˚C; **D**: pH 10.0, T=45˚C.

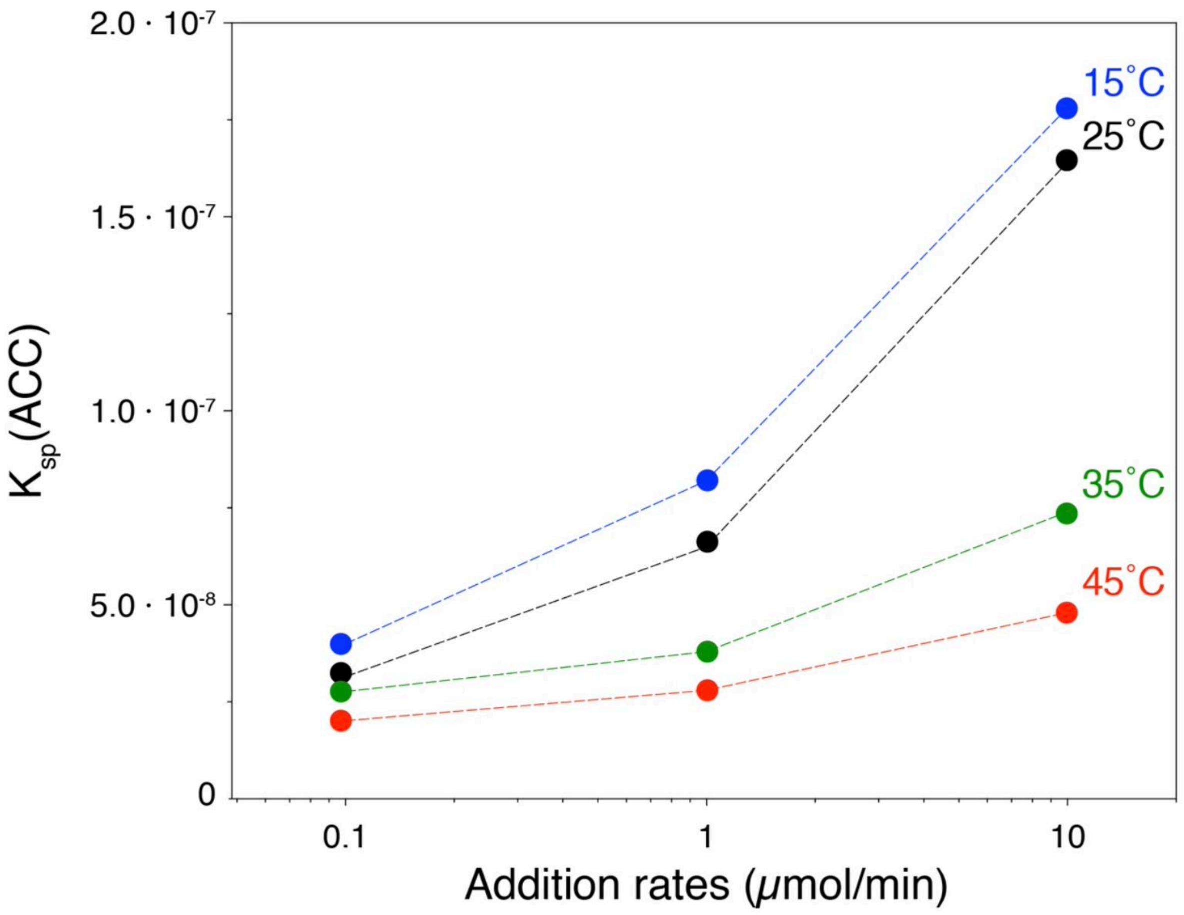

Figure S3. Rate dependent potentiometric titrations at pH 9.0 (also see Fig. S2). With increasing mixing rate, the increasing solubilities of the formed ACCs reflect the higher metastability of the liquid precursors.

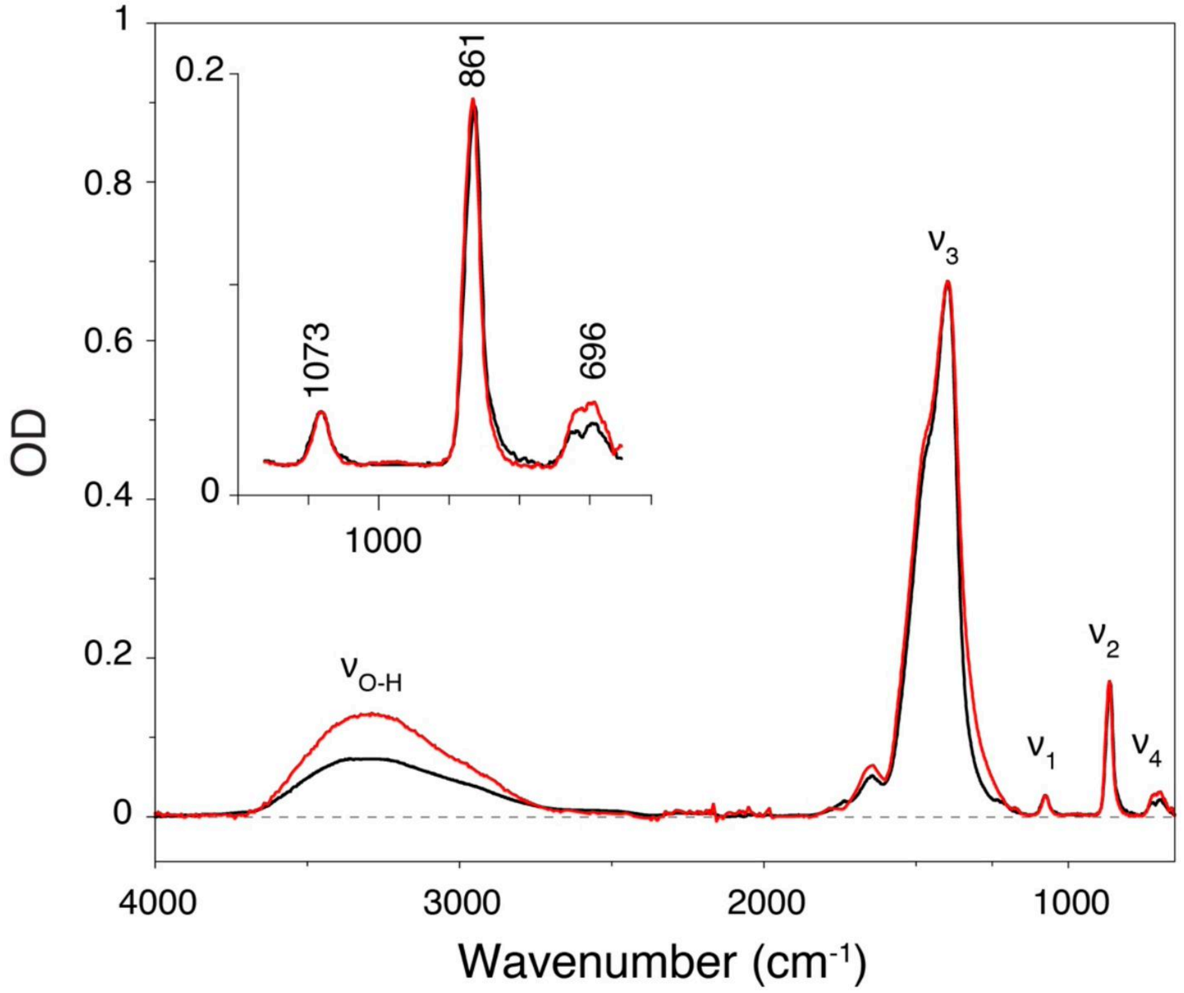

Figure S4. ATR-FTIR spectra of pc-ACC formed for different addition rates, 10 µmol min^{-1} (red) and 0.1 µmol min^{-1} (black). The ν_1, ν_2, ν_3 and ν_4 vibrational modes of the carbonate ion correspond to the symmetric stretching, out of plane bending, asymmetric stretch vibrations and in plane bending, respectively, and the $\nu_{O\text{-}H}$ mode to the O-H water stretching vibration. The proto-structure remains unaffected by the addition rate,[9] but the water content increases.

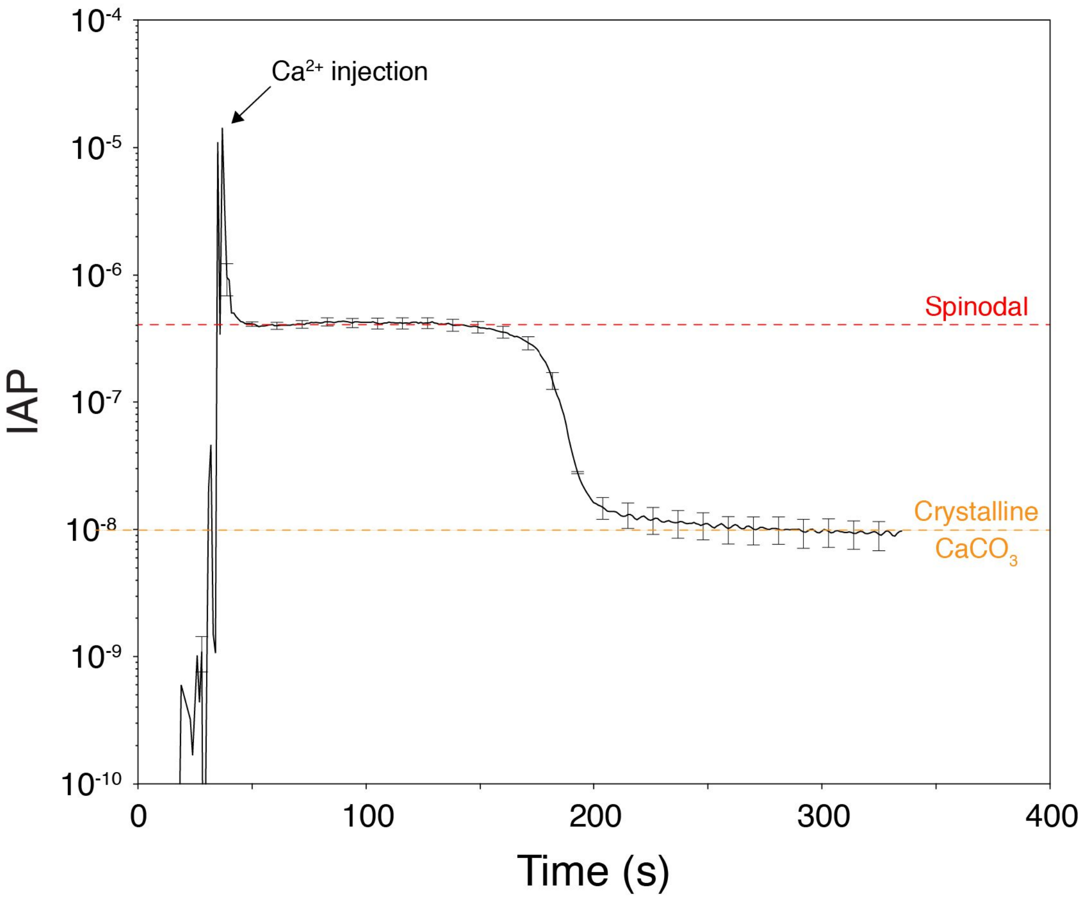

Figure S5. Example time development of the IAP obtained from direct mixing of calcium chloride (0.4M) and pure sodium carbonate solution (0.4M) at a 1:1 volume ratio. The IAP profiles present a strong increase right after mixing and rapidly decrease to stabilise towards an ACC solubility plateau characteristic of the spinodal limit. After an induction time, the IAP drops to a second plateau due to the complete transformation of the ACC precursor. The corresponding temperature dependent data is compiled in Table S3.

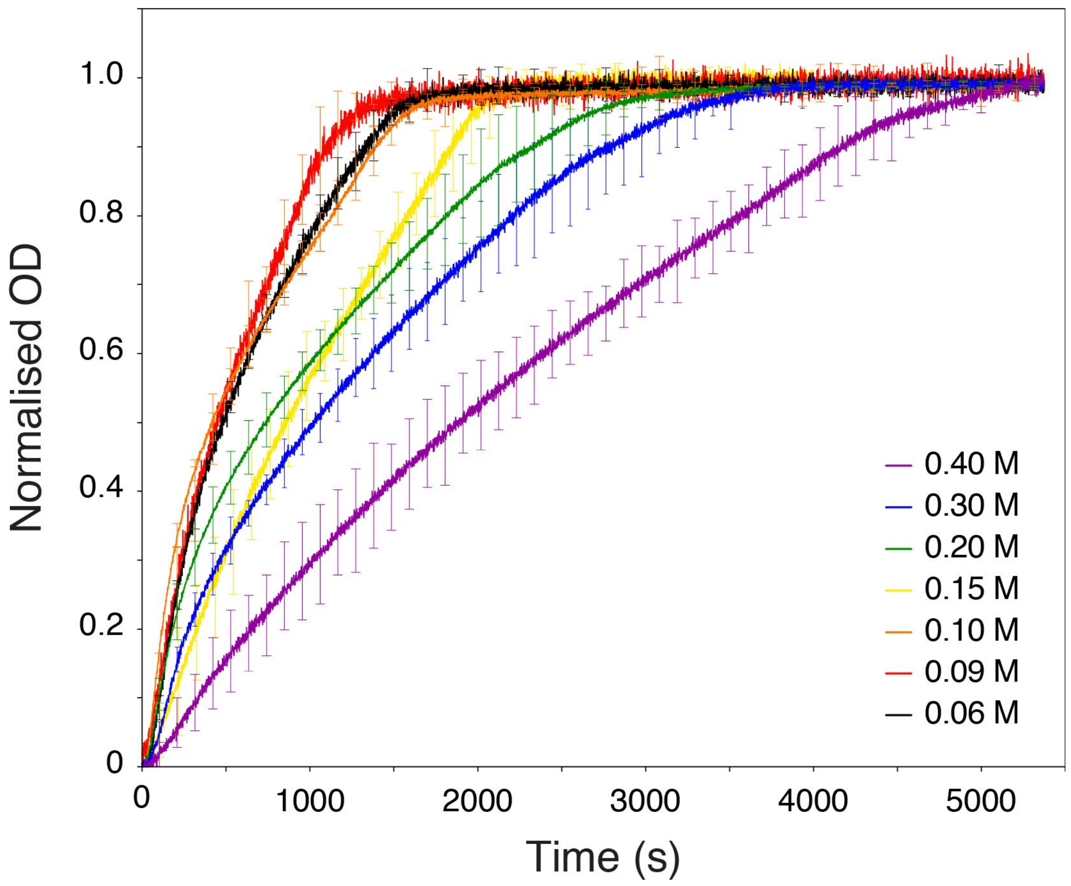

Figure S6. Evolution of the optical density (OD) over time for the carbonate out-of-plane vibrational band (ν_2) at 25˚C after direct mixing of calcium and carbonate solutions with the indicated starting concentrations (at a 1:1 volume ratio). For each starting concentration (i.e. prior mixing), three replicated measurements were normalised and averaged.

Supplementary Tables

Table S1: Ion activity products (IAP) defining the liquid-liquid binodal limit for pH 9.00 and pH 10.0 at 15-45 °C. Values are given as means of N=3 repetitions, SD is the corresponding standard deviation.

**Temperature (˚C)**	**IAP pH 9.00 ($\cdot 10^{-8}$ M^2)**	**± SD ($\cdot 10^{-10}$ M^2)**	**IAP pH 10.0 ($\cdot 10^{-8}$ M^2)**	**± SD ($\cdot 10^{-10}$ M^2)**
15	4.00	5.37	5.08	8.19
20	3.36	5.74	4.21	4.43
25	3.15	2.18	3.86	3.84
30	2.93	8.04	3.41	6.26
35	2.78	3.50	2.78	0.83
40	2.37	3.81	2.26	2.74
45	2.02	6.27	2.04	1.85

Table S2: Standard enthalpies and entropies of PNC formation.

	**ΔS^0(cluster) (JK$^{-1}\cdot$mol^{-1})**	**ΔH^0(cluster) (kJ$\cdot$mol^{-1})**
**pH 9.00**	57.0 ± 8.6	-1.00 ± 0.15
**pH 10.0**	83.0 ± 12.5	7.05 ± 1.05

Table S3: Ion activity product defining the liquid-liquid spinodal limit between 15-45˚C.

Values are given as means of N=3 repetitions, SD is the corresponding standard deviation.

**Temperature (˚C)**	**Liquid-liquid spinodal pH 10.0 ($\cdot 10^{-7}$ M^2)**	**± SD ($\cdot 10^{-7}$ M^2)**
15	4.45	2.07
20	4.20	1.64
25	5.49	2.58
30	3.35	1.92
35	2.56	0.58
40	2.27	1.11
45	2.86	1.13

Supplementary Discussion 1

Proof: Pre-nucleation ion association and consistency of pre-nucleation cluster formation with experimental binding data

Ion association yielding pre-nucleation clusters (PNCs) may be described by an infinite series of inter-connected equilibria;

$Ca^{2+}_{aq}+CO^{2-}_{3,aq}\rightleftharpoons[CaCO_3]^0_{aq}$ with equilibrium constant K(1) eq. 1

$[CaCO_3]^0_{aq}+[CaCO_3]^0_{aq}\rightleftharpoons[CaCO_3]^0_{2,aq}$ with equilibrium constant K(2) eq. 2

$[CaCO_3]^0_{2,aq}+[CaCO_3]^0_{aq}\rightleftharpoons[CaCO_3]^0_{3,aq}$ with equilibrium constant K(3) eq. 3

$[CaCO_3]^0_{3,aq}+[CaCO_3]^0_{aq}\rightleftharpoons[CaCO_3]^0_{4,aq}$ with equilibrium constant K(4) eq. 4

...

The first equilibrium (**Fehler! Verweisquelle konnte nicht gefunden werden.**1) describes simple ion pairing yielding $[CaCO_3]^0$ and the corresponding equilibrium constant K(1) is defined by the law of mass action according to;

$$K(1)=\frac{a\left([CaCO_3]^0_{aq}\right)}{a\left(Ca^{2+}_{aq}\right)\cdot a\left(CO^{2-}_{3,aq}\right)} \qquad \text{eq. 5}$$

where a(i) is the activity of species i defined as;

$$a(i)=\gamma(i)\,\frac{c(i)}{c^0} \qquad \text{eq. 6}$$

with activity coefficient and molar concentration of species i, $\gamma(i)$ and c(i), respectively, and standard concentration c^0=1mol/L. For the dimerization equilibrium of ion pairs (eq. 2), the law of mass action yields;

$$K(2)=\frac{a\left([CaCO_3]^0_{2,aq}\right)}{a\left([CaCO_3]^0_{aq}\right)^2} \qquad \text{eq. 7}$$

The activity of the ion pair in the denominator can be expressed by re-arranging and inserting eq. 5, and we can re-write eq. 7 as;

$$K(2)=\frac{a\left([CaCO_3]^0_{2,aq}\right)}{a\left([CaCO_3]^0_{aq}\right)\cdot K(1)\cdot a\left(Ca^{2+}_{aq}\right)\cdot a\left(CO^{2-}_{3,aq}\right)} \quad \text{eq. 8}$$

The activity of the dimer of ion pairs in the numerator can be expressed by re-arranging and inserting eq. 7, and we obtain from eq. 8;

$$K(2)=\frac{K(2)\cdot a\left([CaCO_3]^0_{aq}\right)}{K(1)\cdot a\left(Ca^{2+}_{aq}\right)\cdot a\left(CO^{2-}_{3,aq}\right)} \quad \text{eq. 9}$$

All subsequent equilibria for the formation of higher associates are of the form;

$$P+M\rightleftharpoons MP \quad \text{eq. 10}$$

where M is the monomeric ion pair and P is a polymer of ion pairs, including the dimer of ion pairs, and the polymer of ion pairs grown by one ion pair MP. Thus, the equilibrium constants K(3), K(4), K(5), ... can be defined as;

$$K(3),K(4),K(5),...=K(P)=\frac{a(MP)}{a(M)a(P)} \quad \text{eq. 11}$$

In this notation, the activity of the ion pair M can be calculated from eq. 5 according to;

$$a(M)=K(1)\cdot a\left(Ca^{2+}_{aq}\right)\cdot a\left(CO^{2-}_{3,aq}\right) \quad \text{eq. 12}$$

whereas the activity of the polymer of ion pairs grown by one ion pair MP is obtained by;

$$a(MP)=K(P\text{-}1)\cdot a(P)a(M) \quad \text{eq. 13}$$

where K(P-1) is the equilibrium constant for the previous association step. By inserting eqs. 12 and 13 into eq. 11, we obtain;

$$K(P)=\frac{K(P\text{-}1)\cdot a(M)}{K(1)\cdot a\left(Ca^{2+}_{aq}\right)\cdot a\left(CO^{2-}_{3,aq}\right)} \quad \text{eq. 14}$$

We recall the assumption that all association steps are equal and independent, as originally introduced in the first publication on PNCs.[2] In the meantime, the validity of this assumption has been corroborated based on computer simulations,[10] and can now also be rationalized mechanistically: since ion association is driven by the entropy gain due to the release of ionic

hydration waters upon calcium and carbonate ions binding together,[11] the thermodynamic driving force should not differ if, e.g., a carbonate ion binds to a single calcium ion, or to a calcium ion that is the end member of a larger, chain-like PNC (the structural form of PNCs is a chain-like, dynamically-ordered liquid-like oxyanion polymer, DOLLOP[10]). In other words, it can be assumed[2,10] **that the *values* of all equilibrium constants K(1), K(2), K(3),... are equal**;

$$K(1)=K(2)=K(3)=...=K(P\text{-}1)=K(P) \qquad \text{eq. 15}$$

Combining eq. 5, eq. 9 and eq. 14, we can thus write with eq. 15;

$$K(1)=K(2)=K(3)=...=\frac{a\left([CaCO_3]^0_{aq}\right)}{a\left(Ca^{2+}_{aq}\right)\cdot a\left(CO^{2-}_{3,aq}\right)} \qquad \text{eq. 16}$$

This shows that as per the assumption made, i.e., the *values* of all equilibrium constants are equal, also the *form* of the law of mass action becomes identical for each association step. Hence, for the situation that all equilibrium constants of consecutive associations of ion pairs have the same equilibrium constant (eq. 15), the ion pairing equilibrium cannot be distinguished from the formation of higher associated states, namely, PNCs. We can thus re-formulate eq. 16, writing;

$$K(\text{cluster})=\frac{a\left([CaCO_3]^0_{\text{cluster},aq}\right)}{a\left(Ca^{2+}_{aq}\right)\cdot a\left(CO^{2-}_{3,aq}\right)} \qquad \text{eq. 17}$$

where K(cluster)=K(1)=K(2)=K(3)=...=K(P-1)=K(P). That is, PNC formation with equal equilibrium constants for all association steps of ion pairs can be described by the generic equilibrium;

$$Ca^{2+}_{aq}+CO^{2-}_{3,aq}\rightleftharpoons[CaCO_3]^0_{\text{cluster},aq} \qquad \text{eq. 18}$$

In the experiments with the calcium-ion selective electrode (ISE), the free calcium concentration upon constant addition of dilute calcium solution into dilute carbonate buffer is monitored, yielding a linear increase in free calcium in the pre-nucleation stage. The

difference between the added calcium and detected free calcium thus yields the bound calcium, which also increases linearly. We then expect a relation of the form;

$$a\left(Ca_{aq}^{2+}\right)=C\cdot a\left(Ca_{bound}^{2+}\right) \qquad \text{eq. 19}$$

where C is a constant and $a(Ca_{bound}^{2+})$ is the activity of bound calcium. For the free calcium activity in dependence of the bound calcium, we may write, using eq. 17;

$$a\left(Ca_{aq}^{2+}\right)=\frac{a\left(Ca_{bound}^{2+}\right)}{K(cluster)\cdot a\left(CO_{3,aq}^{2-}\right)} \qquad \text{eq. 20}$$

This is a linear relationship for the case that $a(CO_{3,aq}^{2-})$=constant, i.e., C=1/[K(cluster) $a(CO_{3,aq}^{2-})$]. This precondition is met within only minor deviations for the titration experiments: In the pre-nucleation stage, the total carbonate concentration, including carbonate, bicarbonate and carbon dioxide, is much larger than the calcium concentration, whereas the pH is kept constant and the fraction of the different species constituting the carbonate buffer consequently does not vary. Thereby, the binding of carbonate ions is buffered by the equilibrium with bicarbonate, and the free carbonate activity remains essentially constant.

Last, it should be noted that the formation of *defined*, neutral clusters with n, and only n, calcium carbonate units does not represent the PNC notion and is inconsistent with the experimentally found, linear binding profiles;

$$nCa_{aq}^{2+}+nCO_{3,aq}^{2-}\rightleftharpoons[CaCO_3]_{n,aq}^{0} \qquad \text{eq. 21}$$

We define the corresponding equilibrium constant K(n);

$$K(n)=\frac{a\left([CaCO_3]_{n,aq}^{0}\right)}{a\left(Ca_{aq}^{2+}\right)^{n}\cdot a\left(CO_{3,aq}^{2-}\right)^{n}}=\frac{a\left(Ca_{bound}^{2+}\right)}{a\left(Ca_{aq}^{2+}\right)^{n}\cdot a\left(CO_{3,aq}^{2-}\right)^{n}} \qquad \text{eq. 22}$$

The dependence between free calcium activity and bound calcium then becomes the non-linear relation;

$$a\left(Ca_{aq}^{2+}\right)=\frac{1}{a\left(CO_{3,aq}^{2-}\right)}\sqrt[n]{\frac{a\left(Ca_{bound}^{2+}\right)}{K(n)}}$$ eq. 23

While the cluster with n constituents forms also in the PNC model, eq. 23 does not account for the convolution of the ion binding with all other association equilibria. Thus, eq. 23 does not invalidate eq. 20. The convolution of ion binding in a series of coexisting clusters yields a linear binding for equal equilibrium constants for each association step (eq. 20).

Supplementary Discussion 2

Pre-nucleation Ion Association Evaluated based on a Multiple Binding Model

As demonstrated previously,[2] the binding of ions in PNCs can be evaluated in more detail based on a model that was originally derived for assessing protein-ligand binding equilibria.[12] The basic assumption is that upon binding calcium ions to a carbonate ion, all possible binding events are equal and independent. This assumption mirrors eq. 15 (see section 1).

The multiple-binding equilibrium is formulated for the binding of calcium ions on a carbonate ion, because carbonate ions are present in excess in typical titration experiments, serving as a 'lattice' for calcium binding, and can thus be written as;

$$CO_{3,aq}^{2-} \xleftrightarrow{+Ca_{aq}^{2+}} [CaCO_3]_{aq}^{0} \xleftrightarrow{+Ca_{aq}^{2+}} [Ca_2CO_3]_{aq}^{2+} \xleftrightarrow{+Ca_{aq}^{2+}} \ldots \qquad \text{eq. 24}$$

This model is considered a *microscopic* model for the binding of calcium ions to a carbonate ion, where the calcium ions may or may not have already bound another carbonate ion, due to the basic assumption of equal and independent binding made. It can thus only assess the number of binding sites x for calcium ions on a carbonate ion, which must be larger than one if clusters can form. The formed clusters of ion pairs can still be macroscopically uncharged, consistent with the experimentally observed ratio of bound calcium to bound carbonate of 1:1,[2] because the calcium ions binding to the carbonate ion considered within a microscopic perspective may already have bound another carbonate ion, and the model is thus consistent with the macroscopic polymerization of ion pairs discussed in the previous section. As shown in detail elsewhere,[2,12] the model yields;

$$1+\frac{n\left(CO_{3,aq}^{2-}\right)}{n\left(Ca_{bound}^{2+}\right)}=\frac{1}{x}+\frac{1}{x\cdot K_{mb}}\cdot\frac{1}{a\left(Ca_{aq}^{2+}\right)} \qquad \text{eq. 25}$$

Here, n(i) denotes the moles of species i. A plot of the left-hand side of eq. 25 versus the reciprocal calcium activity yields a straight line, where the ordinate intercept gives the reciprocal number of binding sites 1/x and the slope gives the reciprocal product of binding sites and corresponding microscopic equilibrium constant $1/(xK_{mb})$. Comparison of eq. 17 and eq. 25 shows that the microscopic binding parameters yield the macroscopic association constant (eq. 17) according to;

$$K(\text{cluster}) = x \cdot K_{mb} \qquad \text{eq. 26}$$

In other words, the microscopic multiple-binding model 'subdivides' the macroscopically observed binding into dimerization and an additional contribution of further association steps (SI section 1, eqs. 1-4), which, in this microscopic view, become evident in a number of binding sites x>1, which are energetically equivalent with the first association step (eq 1). The x-parameter is thereby defined as the number of binding sites for calcium ions on carbonate ions (eq. 25) but since the same amounts of calcium and carbonate ions are bound in PNCs,[2,11] it follows that the number of binding sites for carbonate ions on calcium ions must be identical. Since this microscopic x-parameter thus represents the average number of binding sites for all calcium and carbonate ions present in the solution, the fraction of bound ions f has to be taken into account in order to calculate the calcium-carbonate coordination number only *within* PNCs from the x parameter;[10]

$$f = \frac{n\left(Ca^{2+}_{bound}\right)}{n\left(Ca^{2+}_{bound}\right) + n\left(Ca^{2+}_{free}\right)} \qquad \text{eq. 27}$$

The average coordination number N of calcium by carbonate (and vice versa) for ions bound in PNCs can then be obtained from;

$$N = \frac{x}{f} \qquad \text{eq. 28}$$

Experimentally and computationally, N=2 within experimental accuracy, which is consistent with a chain-like structural form of PNCs called a 'dynamically-ordered liquid-like oxyanion polymer' (DOLLOP).[10]

The corresponding Gibbs standard free energy of PNCs can thus be obtained by;

$$\Delta G^0(\text{cluster}) = -RT \cdot \ln K(\text{cluster}) \qquad \text{eq. 29}$$

with absolute temperature *T* and the universal gas constant R.

Similarly, Gibbs standard free energy corresponding to the microscopic binding of calcium and carbonate only, without PNC formation, can be obtained by;

$$\Delta G^0(\text{micro}) = -RT \cdot \ln K_{mb} \qquad \text{eq. 30}$$

Supplementary Discussion 3

Prediction of spinodal and binodal limits based on pre-nucleation ion association thermodynamics

As described in Supplementary Discussions 1, the PNC model assumes that all equilibrium constants of successive association equilibria (eqs. 1-4) have equal values. The standard free energy of the ion pair (eqs. 1 and 6) can thus be calculated according to;

$$\Delta G^0\left(CaCO^0_{3,aq}\right)=-RT\cdot \ln K(1)=-RT\ln K(\text{cluster})=\Delta G^0(\text{cluster}) \qquad \text{eq. 31}$$

where $\Delta G^0(i)$ is the standard free energy of species i, R is the universal gas constant, and T the absolute temperature. As per eq. 16, the individual standard free energies of all associated states are equal, and this value thus represents an average over all associated states (eq. 16), $\Delta G^0(\text{cluster})$. However, this does not represent the gross stability of individual clusters, i.e. when formed from the monomeric ions. For the formation of the dimer of ion pairs directly from the free ions, we can write;

$$2Ca^{2+}_{aq}+2CO^{2-}_{3,aq}\rightleftharpoons[CaCO_3]^0_{2,aq} \qquad \text{eq. 32}$$

and, with it, define;

$$K(2_{direct})=\frac{a\left([CaCO_3]^0_{2,aq}\right)}{\left[a\left(Ca^{2+}_{aq}\right)\cdot a\left(CO^{2-}_{3,aq}\right)\right]^2} \qquad \text{eq. 33}$$

By re-arranging and inserting eqs. 5 and 7 in the denominator and numerator of eq. 33, respectively, we realize with eq. 17;

$$K(2_{direct})=K(1)\cdot K(2)=K(\text{cluster})^2 \qquad \text{eq. 34}$$

and for the corresponding standard free energy of the dimer of ion pairs;

$$\Delta G^0\left([CaCO_3]^0_{2,aq}\right)=-2RT\cdot \ln K(\text{cluster}) \qquad \text{eq. 35}$$

Due to eqs. 11-17, we can generally express the standard free energy of a specific PNC consisting of m ion pairs as;

$$\Delta G^0([CaCO_3]^0_{m,aq})=-mRT\cdot lnK(cluster) \quad \text{eq. 36}$$

This shows that the PNCs become, considerably, more and more stable with increasing cluster size m. Owing to the properties of the law of mass action, however, the larger clusters will still be less and less abundant than the smaller ones, *but only at low IAPs*. A direct consequence is that the interconnected PNC equilibria break down at a specific IAP, at which the activity of the species of a particular higher association step becomes larger than that of the previous one. In this case, every higher associated state draws the available substance from all previous association equilibria and phase separation will occur spontaneously, based on ion association. This is spinodal liquid-liquid demixing. Because all equilibria subsequent to the first association steps (eqs. 1-4) have the same form (eqs. 11-17), this is the case for;

$$a([CaCO_3]^0_{2,aq})\geq a([CaCO_3]^0_{aq}) \quad \text{eq. 37}$$

Re-arranging eq. 5 and inserting the activity of the ion pair in the denominator of eq. 8 yields with eq. 16;

$$K(2)=\frac{a([CaCO_3]^0_{2,aq})}{[K(cluster)\cdot IAP]^2}=\frac{a([CaCO_3]^0_{aq})}{IAP} \quad \text{eq. 38}$$

Solving eq. 38 for IAP yields;

$$\frac{a([CaCO_3]^0_{2,aq})}{a([CaCO_3]^0_{aq})[K(cluster)]^2}=IAP \quad \text{eq. 39}$$

We define the specific IAP, above which eq. 37 becomes true, as IAP(spinodal), and with $a([CaCO_3]^0_{2,aq})=a([CaCO_3]^0_{aq})$ as the limiting case eq. 39 gives;

$$IAP(spinodal)=\frac{1}{[K(cluster)]^2} \quad \text{eq. 40}$$

At the binodal limit, liquid-liquid phase separation from PNC precursors is a question of the probability of a structural change occurring within PNCs from a mechanistic point of view. At

the same time, it is a question of the probability for the direct formation of the different polymorphs, p(polymorph), versus the probability of liquid-liquid demixing, p(demix). Hence, both parameters have to be assessed as a function of the IAP of a given solution. From the viewpoint of Boltzmann statistics, we may write;

$$\mathrm{p(polymorph)} \propto \exp(-\Delta G^0\mathrm{(polymorph)}/RT) \qquad \text{eq. 41}$$

where ΔG^0(polymorph) is the standard free energy of calcite, aragonite, or vaterite that can be calculated from the corresponding solubility K_{sp}(polymorph) according to;

$$\Delta G^0\mathrm{(polymorph)} = -RT\cdot\ln\left[\frac{1}{K_{sp}\mathrm{(polymorph)}}\right] \qquad \text{eq. 42}$$

Combination of eqs. 41 and 42 yields;

$$\mathrm{p(polymorph)} = Y\cdot\frac{1}{K_{sp}\mathrm{(polymorph)}} \qquad \text{eq. 43}$$

where the parameter Y is a (unknown) function f of the actual IAP in the mother liquid, f(IAP). As shown in previous work, at the liquid-liquid binodal limit, the barrier for the direct nucleation of crystals is formidable,[8] and hence, the probability p(polymorph) is essentially zero;

$$0 \approx Y\cdot\frac{1}{K_{sp}\mathrm{(polymorph)}} \qquad \text{eq. 44}$$

Given the values of K_{sp}(polymorph), with regards to the function Y, we can thus conclude that at the liquid-liquid binodal limit;

$$0 < Y = f(\mathrm{IAP}) \ll 1 \qquad \text{eq. 45}$$

In analogy with eq. 41, we can express the probability for liquid-liquid demixing as;

$$\mathrm{p(demix)} \propto \exp(-\Delta G^0\mathrm{(l\text{-}l)}/RT) \qquad \text{eq. 46}$$

where ΔG^0(l-l)=-RTlnK(l-l) is the standard free energy of liquid-liquid demixing with corresponding equilibrium constant K(l-l). As noted previously,[8] the fact that there is no discontinuity or kink in the pre-nucleation ion binding upon liquid-liquid demixing implies that the values of K(l-l)=K(cluster). We can thus rewrite eq. 46;

$$\mathrm{p(demix)=X\cdot K(cluster)} \qquad \text{eq. 47}$$

where X is another unknown function g of the IAP, g(IAP). Again, previous work[8] showed that liquid-liquid demixing occurred readily upon crossing the liquid-liquid binodal limit, at least at sufficiently slow mixing rates, that is, there is no major barrier associated with this event, and at the binodal limit, p(demix)≈1. We can thus re-write eq. 47;

$$\mathrm{0=lnX+lnK(cluster)} \qquad \text{eq. 48}$$

Thus, at the liquid-liquid binodal limit, eqs. 48 and 44 are approximately equal;

$$\mathrm{lnX + lnK(cluster) \approx Y\frac{1}{K_{sp}(polymorph)}} \qquad \text{eq. 49}$$

Both sides of eq. 49 are approximately zero at the binodal limit. In the following, we thus neglect a possible contribution of infinitesimals of different orders, which might cause that eq. 49 does not hold exactly. All of the following depends on this assumption. Re-arranging eq. 49 yields;

$$\mathrm{lnX\cdot K_{sp}(polymorph)=Y\text{-}K_{sp}(polymorph)lnK(cluster)} \qquad \text{eq. 50}$$

Using eq. 45, eq. 50 can be simplified;

$$\mathrm{lnX\cdot K_{sp}(polymorph)=\text{-}K_{sp}(polymorph)lnK(cluster)} \qquad \text{eq. 51}$$

A trivial solution of equation 51 is obviously lnX = -lnK(cluster). Still, the left-hand-side of eq. 51 represents the product of a (unknown) function of the IAP in the mother solution, lnX, and the solubility of the given polymorph, which represents an ion activity product itself. Eq.

51 is valid only at the binodal limit. Thus, we propose that the left-hand-side of eq. 51 can be interpreted to be proportional to the specific ion activity product defining the liquid-liquid binodal limit, IAP(binodal), and we obtain;

$$\mathrm{IAP(binodal)} \propto \mathrm{K_{sp}(polymorph)\cdot lnK(cluster)} \qquad \text{eq. 52}$$

Thus, we propose that the liquid-liquid binodal limit is given by;

$$\mathrm{IAP(binodal)=A(polymorph)\cdot K_{sp}(polymorph)\cdot lnK(cluster)} \qquad \text{eq. 53}$$

where A(polymorph) is a (a priori unknown) constant. We stress that eq. 53 has not been derived definitely here, and relies on the assumptions and interpretations outlined above. Eq. 53 poses a conjecture—which has its foundation in our assessment of the probability of liquid-liquid demixing versus that of the direct formation of crystalline polymorphs—rather than an unambiguously proven equation.

The dense liquid adjusts its composition according to that of the mother liquid; the underlying equilibrium between mother liquid L1 and dense liquid L2 can be formally written as;

$$\left[\mathrm{Ca^{2+}_{aq}+CO^{2-}_{3,aq}}\right]_{L1} \rightleftharpoons \left[\mathrm{Ca^{2+}_{aq}+CO^{2-}_{3,aq}}\right]_{L2} \qquad \text{eq. 54}$$

With;

$$\mathrm{K(l\text{-}l)}=\frac{\mathrm{a\left(Ca^{2+}_{aq}\right)_{L2}a\left(CO^{2-}_{3,aq}\right)_{L2}}}{\mathrm{a\left(Ca^{2+}_{aq}\right)_{L1}a\left(CO^{2-}_{3,aq}\right)_{L1}}}=\frac{\mathrm{IAP(L2)}}{\mathrm{IAP(L1)}} \qquad \text{eq. 55}$$

Macroscopically, this equilibrium is indistinguishable from ion association yielding PNCs (eq. 17), as the dense liquid droplets formally belong to a new phase but are structurally very similar to PNCs.[10] Since no change in pre-nucleation slope is observed upon liquid-liquid separation (also see above and eqs. 46 and 47), the value of the corresponding equilibrium constant K(l-l) is identical with that of K(cluster),[13]

$$K(\text{l-l}) = \frac{IAP(L2)}{IAP(L1)} = K(\text{cluster}) \quad \text{eq. 56}$$

At the critical point, IAP(L1)=IAP(L2), i.e. K(cluster)=1. The critical temperature $T_{crit.}$ can thus be calculated using;

$$\Delta G^0(\text{cluster}) = -RT\ln 1 = 0 = \Delta H^0(\text{cluster}) - T_{crit.}\Delta S^0(\text{cluster}) \quad \text{eq. 57}$$

that is;

$$T_{crit.} = \frac{\Delta H^0(\text{cluster})}{\Delta S^0(\text{cluster})} \quad \text{eq. 58}$$

Notably, the spinodal limit becomes IAP(spinodal)=1 at $T_{crit.}$ (eq. 40), which is a lower critical solution temperature, categorically. That is, phase separation via spinodal demixing of PNCs cannot occur below $T_{crit.}$, because higher associated states are inherently unstable in this temperature regime, where K(cluster)<1 and ΔG^0(cluster)>0, for any IAP below the critical temperature. Consistently, the binodal limit vanishes at the critical temperature, where lnK(cluster)=ln1=0 (eq. 53).

References

1. Kellermeier, M., Picker, A., Kempter, A., Cölfen, H. & Gebauer, D. A straightforward treatment of activity in aqueous $CaCO_3$ solutions and the consequences for nucleation theory. *Adv. Mater.* **26**, 752–757 (2014).

2. Gebauer, D., Völkel, A. & Cölfen, H. Stable prenucleation calcium carbonate clusters. *Science* **322**, 1819–1822 (2008).

3. Hauser, K., Krejtschi, C., Huang, R., Wu, L. & Keiderling, T. A. Site-Specific Relaxation Kinetics of a Tryptophan Zipper Hairpin Peptide Using Temperature-Jump IR Spectroscopy and Isotopic Labeling. *J. Am. Chem. Soc.* **130**, 2984–2992 (2008).

4. Krejtschi, C. & Hauser, K. Stability and folding dynamics of polyglutamic acid. Eur *Biophys J* DOI 10.1007/s00249-011-0673-8, 673–685 (2011).

5. Krüger, A. et al. Interactions of p53 with poly(ADP-ribose) and DNA induce distinct changes in protein structure as revealed by ATR-FTIR spectroscopy. *Nucleic Acids Res.* **47**, 4843–4858 (2019).

6. Meyer, P. S., Yung, J. W. & Ausubel, J. H. A Primer on Logistic Growth and Substitution. *Technol. Forecast. Soc. Change* **61**, 247–271 (1999).

7. Cartwright, J. H. E., Checa, A. G., Gale, J. D., Gebauer, D. & Sainz-Díaz, C. I. Calcium carbonate polyamorphism and its role in biomineralization: how many amorphous calcium carbonates are there? *Angew. Chem. Int. Ed.* **51**, 11960–11970 (2012).

8. Sebastiani, F. et al. Water Dynamics from THz Spectroscopy Reveal the Locus of a Liquid-Liquid Binodal Limit in Aqueous $CaCO_3$ Solutions. *Angew. Chem. Int. Ed.* **56**, 490–495 (2016).

9. Gebauer, D. et al. Proto-calcite and proto-vaterite in amorphous calcium carbonates. *Angew. Chem. Int. Ed.* **49**, 8889–8891 (2010).

10. Demichelis, R., Raiteri, P., Gale, J. D., Quigley, D. & Gebauer, D. Stable prenucleation mineral clusters are liquid-like ionic polymers. *Nat. Commun.* **2**, 590 (2011).

11. Kellermeier, M. et al. Entropy Drives Calcium Carbonate Ion Association. *ChemPhysChem* **17**, 3535–3541 (2016).

12. Scatchard, G. The attractions of proteins for small molecules and ions. *Ann. N.Y. Acad. Sci.* **51**, 660–672 (1949).

13. Sebastiani, F. et al. THz-Spektroskopie erlaubt Rückschlüsse auf die Wasserdynamik und die Lage einer flüssig-flüssig-binodalen Grenze in wässrigen $CaCO_3$-Lösungen. *Angew. Chem.* 1–7 (2016). doi:10.1002/ange.201610554
